# Organotherapy

**Published:** 1905-12

**Authors:** 


**Organotherapy.** By H. Batty Shaw, M.D. Pp. x., 256. London: Cassell & Company. 1905.

The sensational claims of over-zealous followers of the early teaching of Brown-Séquard has undoubtedly hindered the development of what bids fair to be a very important field of therapeutics. The great triumph which the thyroid treatment of myxœdema has justly secured makes the failure of reasonable expectations in other directions the more disappointing. This book gives an account of the physiology of the glands of the body with special reference to internal secretion, and it supplies a review of the practical applications in disease of the derivatives of various organs. Until recently the so-called ductless glands have been considered to be of little importance, but the phenomena of athyrœa and hyperthyrœa in association with myxœdema and Graves's disease have given the subject a vastly increased importance, and have suggested the idea that the products of other organs may be of similar importance in the etiology and treatment of many other diseases.

The first seven chapters are on questions relating to the thyroid and parathyroid glands, and here the author is on safe ground as regards the utility of thyroid preparations in myxœdema, but when we come to the antithesis of this disease he is able to adduce little evidence that anything as yet suggested may be looked upon as a specific remedy. There are few diseases for which thyroid extract has not been tried, and the author gives an excellent summary with abundant references showing what a large amount of investigation has been carried on, but with little result other than in the condition believed to be athyrœa.

Again, with regard to the supra-renal glands, five chapters on this topic give valuable information as to the multiple uses of adrenalin; but as regards the use of supra-renal extract in Addison's disease, there seems to be little reliable evidence of its utility.

The author gives an excellent summary of a vast amount of work done on many other organs, and he takes a very fair judicial view of the evidence before us; his book is one to be thoughtfully consulted and studied, and the serious inquirer will not go away empty.

